# Body Composition in Adults Born at Very Low Birthweight—A Sibling Study

**DOI:** 10.1111/ppe.13147

**Published:** 2025-01-08

**Authors:** Samuel Sandboge, Juho Kuula, Helena Hauta‐alus, Nina Kaseva, Laura Jussinniemi, Johan Björkqvist, Petteri Hovi, Johan G. Eriksson, Outi Mäkitie, Kirsi H. Pietiläinen, Eero Kajantie

**Affiliations:** ^1^ Population Health Unit Finnish Institute for Health and Welfare Helsinki Finland; ^2^ Psychology/Welfare, Faculty of Social Sciences University of Tampere Tampere Finland; ^3^ Department of Radiology, Medical Imaging Center University of Helsinki and Helsinki University Hospital Helsinki Finland; ^4^ Children's Hospital, and Pediatric Research Center University of Helsinki and Helsinki University Hospital Helsinki Finland; ^5^ Clinical Medicine Research Unit University of Oulu Oulu Finland; ^6^ Research Program for Clinical and Molecular Metabolism (CAMM), Faculty of Medicine University of Helsinki Helsinki Finland; ^7^ Department of General Practice and Primary Health Care University of Helsinki and Helsinki University Hospital Helsinki Finland; ^8^ Folkhälsan Research Center Helsinki Finland; ^9^ Department of Obstetrics and Gynecology and Human Translational Research Programme, Yong Loo Lin School of Medicine National University of Singapore Singapore City Singapore; ^10^ Institute for Human Development and Potential (IHDP) Agency for Science Technology and Research (A*STAR) Singapore City Singapore; ^11^ Department of Molecular Medicine and Surgery, Karolinska Institutet, and Clinical Genetics Karolinska University Hospital Stockholm Sweden; ^12^ Obesity Research Unit, Research Program for Clinical and Molecular Metabolism, Faculty of Medicine University of Helsinki Helsinki Finland; ^13^ HealthyWeightHub, Endocrinology, Abdominal Center Helsinki University Hospital and University of Helsinki Helsinki Finland; ^14^ Department of Clinical and Molecular Medicine Norwegian University of Science and Technology Trondheim Norway

**Keywords:** body composition, cohort, DXA, preterm, sibling study, very low birth weight

## Abstract

**Background:**

Individuals born preterm at very low birthweight (VLBW, < 1500 g) tend to attain a smaller adult body size compared with term‐born peers but less is known regarding specific body composition characteristics.

**Objectives:**

We aimed to assess whether adults born at VLBW have less beneficial body composition characteristics, potentially mediating the association between VLBW birth and cardiometabolic disease. Sibling controls were used to account for the potential influence of shared genetic and/or lifestyle factors.

**Methods:**

This cohort study featured 77 adults born at VLBW and 70 term‐born siblings. Dual‐energy X‐ray absorptiometry assessment took place at a mean age of 29 years. Fat mass (FM) distribution was calculated by dividing appendicular by truncal FM. Appendicular skeletal mass (ASM) measurements were used to calculate two indices: Skeletal mass index (SMI, ASM divided by height squared) and ASM/BMI (ASM divided by body mass index). Data were analysed by linear mixed models. An exploratory analysis subdivided the VLBW group by size at gestational age [small or appropriate for gestational age (SGA, defined as a birthweight < 2 SD, or AGA)].

**Results:**

Participants born at VLBW were lighter (−4.7 kg, 95% CI −8.2, −1.2) and shorter (−4.3 cm, 95% CI −6.2, −2.4) than sibling peers. After controlling for sex, age, and maternal factors, they had lower limb/trunk fat ratios (−0.06, 95% CI −0.11, −0.003), LBM (−2.02 kg, 95% CI −3.92, −0.12), ASM (−1.22 kg, 95% CI −2.14, −0.30) and ASM/BMI (−0.05, 95% CI −0.10, −0.004). FM and SMI did not differ between groups. In the subgroup analysis, findings were limited to those born VLBW + SGA.

**Conclusions:**

Individuals born at VLBW had, on average, lower limb/trunk fat ratios and lower relative ASM compared with term‐born siblings. A more centralised fat distribution, as well as lower appendicular muscle mass, could potentially mediate the association between VLBW birth and cardiometabolic risk.

## 1 Background

Preterm birth (< 37 gestational weeks) at very low birthweight (VLBW, < 1500 g) is associated with an increased risk of adult cardiometabolic disease [1, 2, 3, 4, 5]. Individuals born preterm at VLBW tend to have shorter adult height and lower lean body mass (LBM) compared to term‐born peers [6, 7], whereas body mass index (BMI)—a widely used risk factor for cardiometabolic disease [8]—is largely similar between VLBW and term‐born adults [9, 10]. Individuals born small for gestational age (SGA) have an increased risk of central adiposity during childhood [11], and display increased fat mass (FM) and abdominal fat in adulthood [12]. The VLBW and SGA categories overlap and consequently, individuals born VLBW + SGA may be especially predisposed to develop disadvantageous body composition characteristics.

We recently published data on 78 adults born at VLBW with 72 term‐born sibling controls [13], and in contrast to smaller studies with unrelated controls [14], found no differences in abdominal adipose tissue volumes or ectopic fat in liver or bone marrow, measured by magnetic resonance imagining (MRI) and MRI spectroscopy at mean age 29 years. Indeed, it may be that previously observed differences in body composition could, at least partly, be explained by shared familial confounding, something the current study design is able to account for in a novel manner. This study's aim was to compare anthropometric and dual X‐ray absorptiometry (DXA) measurements in the same VLBW and sibling control population and additionally, to explore the potential impact of being born both VLBW and SGA on adult body composition.

## 2 Methods

### 2.1 Participants

**FIGURE 1:**
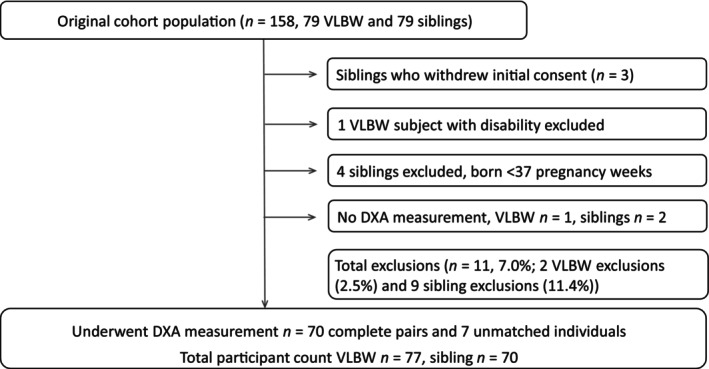
Flow chart of participants.

The study population, described elsewhere in detail [15, 16], was recruited between 2014 and 2017 and originally consisted of 79 individuals born at VLBW and their term‐born siblings. The current study comprises 77 individuals born at VLBW and 70 sibling controls (Figure 1).

### 2.2 Exposure

Information on maternal pregnancy smoking status, weight and age as well as gestational duration, information on gestational hypertension, pre‐eclampsia, proteinuria and birthweight were collected from the maternity clinic and hospital records. We defined SGA as birthweight < −2 SD based on Finnish birthweight references [17], according to Finnish clinical practice. Information on parental education was collected using a standardised questionnaire [15].

### 2.3 Outcomes

Anthropometric and DXA examinations (Hologic Discovery A) were performed at a mean age of 29 years. DXA provided measurements (in kg) and percentages of LBM and FM. Height‐adjusted LBM was calculated separately for men and women. Fat mass index (FMI) was calculated by dividing FM by height squared (kg/m^2^). FM distribution was assessed as limb/trunk fat ratio, calculated by dividing appendicular by truncal FM. ASM was calculated by subtracting appendicular bone mineral content from appendicular lean mass. We also calculated two indices: SMI (ASM divided by height squared, kg/m^2^) and ASM/BMI [ASM divided by BMI, kg/(kg/m^2^)] used for assessing sarcopenia [18, 19]. Applying suggested cut‐off points (SMI < 5.67 and < 7.23 kg/m^2^ [20], and ASM/BMI < 0.512 and < 0.789 [21] in men and women, respectively), we created dichotomous outcome variables to assess the prevalence of reduced muscle mass.

### 2.4 Statistical Analysis

Between‐sibling mean differences with 95% confidence intervals (CI) were calculated using paired *t*‐tests. Mixed model linear regression, with participants nested within families, provided measures of effect sizes and 95% CIs. Mixed models allow for inclusion of unmatched siblings in the analysis.

We compared outcomes between VLBW participants and sibling controls in a series of mixed linear regression models with sibling‐pair as the random effect and the following fixed effects: model 1 adjusted for sex and age. Model 2 additionally adjusted for maternal factors: gestational hypertension, pre‐eclampsia and isolated proteinuria. We additionally created two dummy variables (VLBW + AGA, VLBW + SGA) and entered these as predictor variables replacing VLBW status yes/no.

No interaction was found between VLBW‐birth and sex for any outcome and accordingly, analyses were run with sexes pooled.

### 2.5 Missing Data and Sensitivity Analysis

DXA‐scans from 4 VBLW participants and 7 sibling controls were incomplete due to large body size. A sensitivity analysis repeated all model 1 analyses excluding these 11 participants. The associations remained similar and hence, all participants were included in the final analysis.

Statistical analyses were performed using SPSS software (IBM SPSS Statistics for Windows, version 29).

### 2.6 Ethics Statement

All participants provided informed consent. The study was approved by the ethics committee at the Hospital District of Helsinki and Uusimaa.

## 3 Results

Table 1 shows participant baseline characteristics. The average age was 29 years; 52.4% (77/147) were women. Table S1 presents descriptive DXA‐derived characteristics by group and Table S2 presents anthropometric and DXA measurements by sex.

**TABLE 1:** Characteristics of study population.

	Term (*n* = 70)	VLBW (*n* = 77)	Sibling pairs (*n* = 70)
			Mean difference (95% CI)
Female/male	36/34	41/36	
Parental factors			
Maternal age at birth, mean (SD), years	30.1 (5.1)	29.7 (4.9)	0.5 (−1.2, 2.1)
Maternal BMI, mean (SD), kg/m^2^	22.6 (4.2)	22.5 (4.1)	0.1 (−1.3, 1.5)
Normotensive pregnancy, *n* (%)	46 (65.7)	50 (64.9)	
Gestational and chronic hypertension, *n* (%)	18 (25.7)	4 (5.2)	
Pre‐eclampsia and superimposed PE, *n* (%)	1 (1.4)	21 (27.3)	
Proteinuria, *n* (%)	5 (7.1)	2 (2.6)	
Maternal smoking during pregnancy, *n* (%)			
Smoking, *n* (%)	11 (14.3)	11 (15.7)	
Non‐smoking, *n* (%)	64 (83.1)	54 (75.7)	
Unknown, *n* (%)	2 (2.6)	6 (8.6)	
Completed education level of at least one parent (%)			
Lower secondary or lower	0		
Higher secondary	38.6		
Tertiary	61.4		
Birth characteristics			
Birthweight, mean (SD), g	3404 (432)	1150 (220)	−2244 (−2364, −2124)
Birthweight SD score, mean (SD)	−0.3 (0.9)	−1.3 (1.6)	−0.9 (−1.3, −0.5)
Length of gestation, mean (SD), w	39.8 (1.3)	29.5 (2.5)	−10.3 (−11.0, −9.8)
Small for gestational age, *n* (%)	2 (2.9)	29 (37.7)	
Adult characteristics			
Age, mean (SD), year	29.2 (5.1)	29.6 (2.8)	0.3 (−0.7, 1.3)
Weight, mean (SD), kg	73.9 (17.1)	69.1 (15.4)	−4.7 (−8.2, −1.2)
Height, mean (SD), cm	172.6 (9.5)	167.6 (9.4)	−4.3 (−6.2, −2.4)
BMI, mean (SD), kg/m^2^	24.6 (4.5)	24.5 (4.7)	−0.3 (−1.5, 0.9)
Waist circumference (SD), cm	84.3 (12.2)	84.4 (13.8)	−0.09 (−3.9, 3.8)
Hip circumference (SD), cm	99.1 (10.2)	97.7 (9.8)	−1.6 (−4.6, 1.5)
Waist/hip ratio (SD)	0.8 (0.1)	0.9 (0.1)	0.01 (−0.01, 0.03)

*Note:* Means and standard deviations for the VLBW and term sibling groups are presented together with between‐sibling mean differences with 95% confidence intervals.

Abbreviations: BMI, body mass index, kg/m^2^; CI, confidence interval; d, days; SD, standard deviation; w, weeks.

**FIGURE 2:**
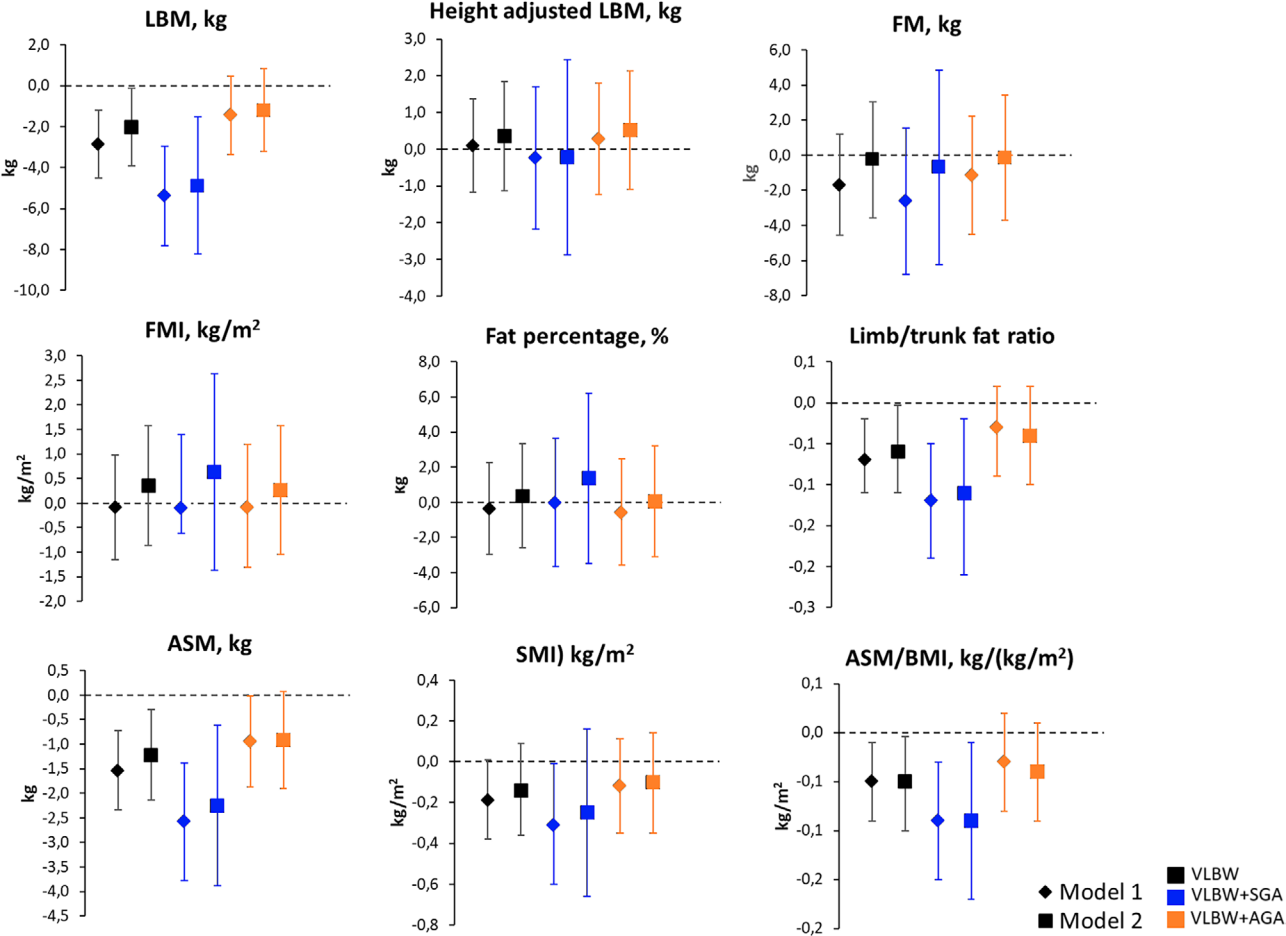
Mean differences (95% CI) between adults born at VLBW, adults born at VLBW + SGA and adults born at VLBW + AGA and term‐born siblings. Mean differences (95% CI error bars) in outcome variables (95% CIs, error bars) in (from left to right) adults born at VLBW, adults born at VLBW and additionally SGA, and adults born at VLBW and additionally AGA, compared to their term‐born siblings (zero line). Model 1 adjusts for sex and age. Model 2 additionally adjusts for maternal gestational hypertension, preeclampsia and isolated proteinuria. AGA, appropriate for gestational age; ASM, appendicular skeletal muscle mass was calculated by subtracting appendicular bone mineral content from appendicular lean mass; ASM/BMI (ASM/BMI); FM, fat mass; FMI, fat mass index (FM/height^2^); LBM, lean body mass; limb/trunk fat ratio was calculated by dividing appendicular by truncal FM; SGA, small for gestational age; SMI, skeletal mass index (ASM/height^2^); VLBW, very low birthweight.

In the fully adjusted model, individuals born VLBW had lower LBM, limb/trunk fat mass ratio, ASM and AMS/BMI index compared to sibling controls (Figure 2 and Table S3). Height‐adjusted LBM, FM and body fat percentage did not differ between groups, however.

Table S4 shows baseline and DXA characteristics for VLBW + SGA and VLBW + AGA subgroups. Compared to controls, VLBW + SGA participants had lower LBM, limb/trunk fat ratio, ASM, SMI and ASM/BMI (Figure 2 and Table S5), whereas the lower ASM among VLBW + AGA participants attenuated in the fully adjusted model (Figure 2 and Table S6).

### 3.1 Comment

We explored the impact of VLBW‐birth on adult body composition. We used same‐sex, term‐born siblings as controls, instead of unrelated individuals, to account for potential familial confounding, that is, shared socio‐economic, environmental and genetic factors. VLBW participants displayed lower LBM, largely explained by their shorter stature, as well as lower average limb/trunk fat mass ratio, indicating more centralised fat distribution which may be less optimal from a cardio‐metabolic perspective [22]. VLBW participants also had lower ASM and ASM/BMI, indicating lower relative appendicular muscle mass. In an exploratory subgroup analysis, findings were restricted to those born VLBW + SGA.

In our previous publication from this cohort featuring DXA measurements, VLBW participants displayed lower femoral neck bone mineral density and lower bone mineral content at all sites compared to their siblings [15]. Differences were primarily explained by the VLBW participants' shorter height and were less pronounced than those of previous studies, indicating a potential influence of shared familial factors, in addition to that of VLBW status.

Few studies have explored the associations between VLBW‐birth and adult body composition. In a study from the Helsinki Study of Very Low Birth Weight Adults featuring 163 individuals aged 18–27 years born at VLBW as well as in a follow‐up study at mean age 36 years additionally featuring participants from the NTNU Low Birth Weight Life cohort, LBM was lower among VLBW participants compared to controls [6]. Consistent with our findings, differences in LBM attenuated after height adjustment [6, 7]. While 22 of our study's VLBW‐born participants, participated in the two aforementioned studies, these did not use sibling controls.

A 2023 meta‐analysis, featuring over 250,000 individuals, demonstrated a positive association between gestational duration and childhood BMI, whereas preterm‐born adolescents (ages 15–19 years) tended to reach similar BMI levels as term‐born peers [23]. The study additionally demonstrated an increased risk of overweight during adolescence among those born very preterm (weeks 28–33). In contrast, we found no differences regarding BMI or waist–hip ratio between VLBW‐born and controls.

In our cohort, 37.7% of the VLBW participants were born SGA, an expected proportion given that birthweights of preterm‐AGA infants often surpass the VLBW‐threshold of 1500 g [24]. Our findings were limited to those born VLBW + SGA, whereas the VLBW + AGA group did not differ from controls. In a study by Hack et al. [9] VLBW + SGA status was similarly disadvantageous regarding adult height and weight, although only in men.

We studied fat distribution using limb/trunk fat mass ratio, suggested to predict cardiometabolic risk independently of BMI [25]. In our study, those born at VLBW had lower ASM and ASM/BMI compared to sibling controls. Measurements of appendicular skeletal muscle mass have been suggested for inclusion in the assessment of sarcopenia [18, 19], a condition associated with increased mortality and disability [26, 27].

The study's strengths include a well‐defined cohort as well as its novel design, using sibling controls instead of unrelated individuals, aiming to account for potential familial confounding. Conversely, the study design and recruitment are associated with potential over‐matching, as siblings with similar personalities and/or behaviour may participate more willingly. Furthermore, individuals born at VLBW without same‐sex siblings and/or with an age difference above 10 years were not considered for inclusion, potentially decreasing generalizability. Outcomes were assessed using DXA, limiting comparison with studies employing other methodologies. Finally, the results from the subgroup analysis should be seen as exploratory and interpreted cautiously, as the study was not powered, nor designed for assessing the impact of VLBW + SGA birth.

## 4 Conclusions

In this study of 77 VLBW‐born adults and 70 sibling controls, those born at VLBW were shorter, lighter and had a lower lean body mass, lower limb/trunk fat mass ratio and lower relative appendicular muscle mass than their term‐born sibling peers. Differences in lean body mass could be explained by the VLBW participants' shorter stature. In an exploratory subgroup analysis, the differences in body composition were restricted to SGA‐born VLBW participants. A centralised fat distribution could, together with decreased appendicular muscle mass, potentially be factors mediating the association between preterm birth at VLBW and cardiometabolic risk. Our findings highlight the importance of taking other features of body composition than BMI and weight into account when assessing cardiometabolic risk in this group of individuals.

## Author Contributions

S.S. participated in data cleaning, processed the data, draughted the initial manuscript and finalised the manuscript. J.K. was the primary agent of the sibling study and was widely responsible for participant recruitment, logistics of the clinical study, and data collection and analysis. H.H. and N.K. contributed to the design of the work, participated in data cleaning, and reviewed and revised the manuscript. L.J. contributed to the design of the work and reviewed and revised the manuscript. J.B. collected data, participated in data cleaning, and reviewed and revised the manuscript. P.H., J.G.E., O.M. and K.H.P. conceptualised and designed the study and critically reviewed the manuscript for important intellectual content. E.K. conceptualised and designed the study, coordinated and supervised data collection, and critically reviewed the manuscript for important intellectual content. All authors approved the final manuscript as submitted and agree to be accountable for all aspects of the work.

## Ethics Statement

The study was approved by the ethics committee at the Hospital District of Helsinki and Uusimaa.

## Consent

All participants provided informed consent.

## Conflicts of Interest

The authors declare no conflicts of interest.

## Supporting information


FIGURE S1.

TABLE S1.

## Data Availability

The data are not publicly available due to privacy or ethical restrictions. Investigators requesting data access should contact the corresponding author (S.S.). Request could be subject to ethics review and/or participant consent.
