# Supplementary figures and images for: Body Composition in Adults Born at Very Low Birthweight—A Sibling Study
Source: Paediatr Perinat Epidemiol. 2025 Jan 8;39(2):177–83. doi: 10.1111/ppe.13147 (PMC11866735; doi:10.1111/ppe.13147)

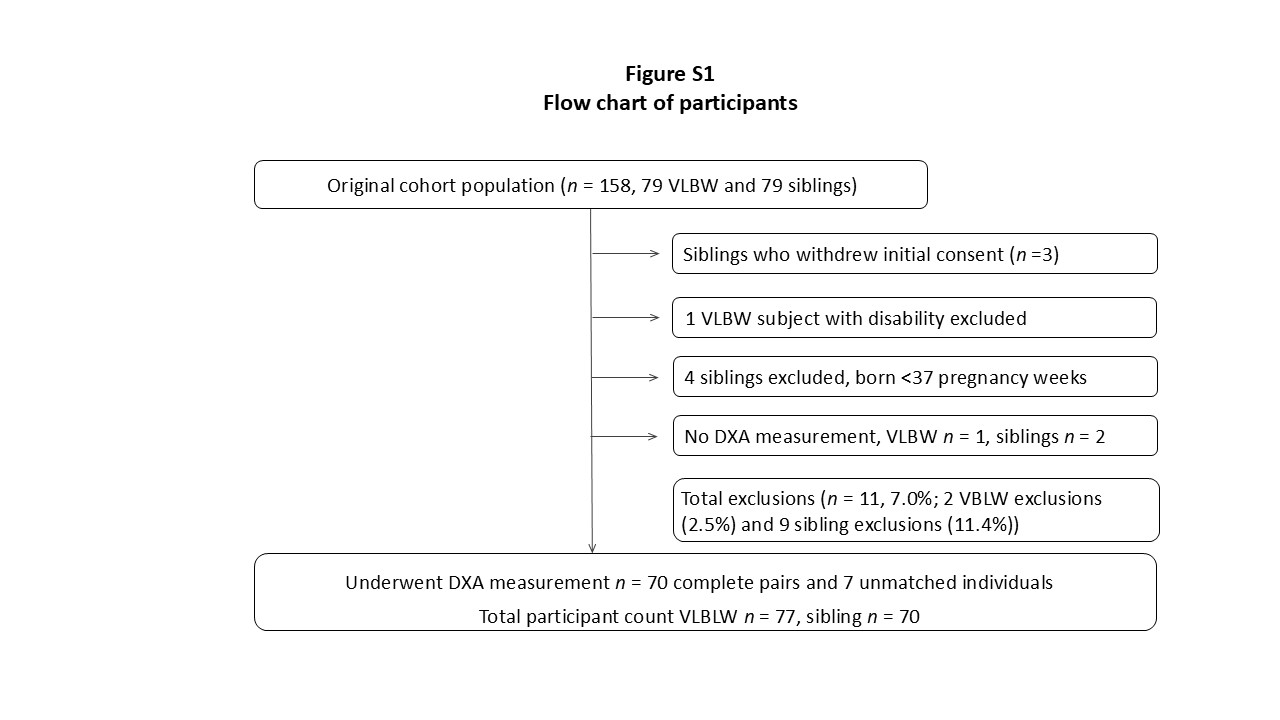

Supplement: Supplementary file 1 — FIGURE S1. [file PPE-39-177-s001.jpg]
