# Supplementary material for: Body Composition in Adults Born at Very Low Birthweight—A Sibling Study
Source: Paediatr Perinat Epidemiol. 2025 Jan 8;39(2):177–83. doi: 10.1111/ppe.13147 (PMC11866735; doi:10.1111/ppe.13147)
Supplement: Supplementary file 2 — TABLE S1. [file PPE-39-177-s002.docx]

**eTable 1 – DXA characteristics for participants born at VLBW and term-born siblings.**

|  | **Term (n = 70)** |  | **VLBW (n = 77)** |  | **Sibling pairs (n = 70)** |
| --- | --- | --- | --- | --- | --- |
|  |  |  |  |  | Mean difference (95% CI) |
|  |  |  |  |  |  |
| Lean body mass, mean (SD), kg | 48.5 (12.3) |  | 45.2 (10.9) |  | -2.8 (-4.5, -1.1) |
| Fat mass, mean (SD), kg | 23.0 (10.6) |  | 21.4 (10.9) |  | -1.9 (-4.9, 1.0) |
| Fat percentage, mean (SD), % | 31.6 (9.9) |  | 31.4 (11.2) |  | -0.7 (-3.5, 2.1) |
| Limb/trunk fat ratio, mean (SD) | 0.92 (0.27) |  | 0.85 (0.25) |  | -0.06 (-0.11, -0.01) |
| Fat mass index (SD), kg/m^2^ | 7.8 (3.6) |  | 7.7 (4.1) |  | -0.2 (-1.3, 0.9) |
| Appendicular skeletal muscle mass (SD), kg | 20.2 (6.0) |  | 18.7 (5.4) |  | -1.5 (-2.4, -0.7) |
| Skeletal muscle mass index (ASM/height^2^; SMI) (SD), kg/m^2^ | 6.7 (1.4) |  | 6.5 (1.3) |  | -0.2 (-0.4, 0.004) |
| Decreased SMI, n (%)^a^ | 31 (44.3) |  | 36 (46.8) |  |  |
| ASM/BMI, mean (SD), kg/(kg/m^2^) | 0.83 (0.20) |  | 0.78 (0.22) |  | -0.05 (-0.09, -0.002) |
| Decreased ASM/BMI, n (%)^b^ | 7 (10) |  | 13 (17.0) |  |  |

Means and standard deviations for the VLBW and term sibling groups are presented together with between-sibling mean differences with 95% confidence intervals.

Abbreviations: CI, confidence interval; SD, standard deviation; FM, fat mass; FMI, fat mass index (FM / height^2^); ASM, Appendicular skeletal muscle mass was calculated by subtracting appendicular bone mineral content from appendicular lean mass; SMI, Skeletal mass index (ASM/height^2^), BMI, body mass index. Limb/trunk fat ratio was calculated by dividing appendicular by truncal FM.

^a^Decreased SMI in women: <5.67 kg/m^2^, in men <7.23 kg/m^2^; ^b^Decreased ASM/BMI women: <0.512, in men <0.789.

**eTable 2: Adult characteristics of study population by sex and birth size.**

|  | **Female (n = 77)** | | | |  | **Male (n = 70)** | | | |
| --- | --- | --- | --- | --- | --- | --- | --- | --- | --- |
|  | **Term (n = 36)** | **n** | **VLBW (n = 41)** | **n** |  | **Term (n = 34)** |  | **VLBW (n = 36)** | **n** |
| Weight, mean (SD), kg | 65.4 (15.2) | 36 | 63.6 (15.4) | 41 |  | 82.9 (14.4) | 34 | 75.3 (13.0) | 36 |
| Height, mean (SD), cm | 165.7 (5.4) | 36 | 162.1 (6.9) | 41 |  | 180.0 (7.0) | 34 | 173.9 (7.8) | 36 |
| BMI, mean (SD), kg/m^2^ | 23.8 (4.9) | 36 | 24.1 (5.4) | 41 |  | 25.5 (3.8) | 34 | 24.9 (3.9) | 36 |
| Waist circumference (SD), cm | 79.0 (11.6) | 29 | 80.3 (14.7) | 33 |  | 89.7 (10.4) | 29 | 88.6 (11.6) | 32 |
| Hip Circumference (SD), cm | 97.2 (11.7) | 29 | 97.5 (12.1) | 32 |  | 101.1 (8.3) | 29 | 97.9 (6.8) | 32 |
| Waist/hip ratio (SD) | 0.8 (0.1) | 29 | 0.8 (0.1) | 32 |  | 0.9 (0.1) | 29 | 0.9 (0.1) | 32 |
| Lean body mass, mean (SD), kg | 39.4 (5.5) | 36 | 37.1 (4.9) | 41 |  | 58.2 (10.0) | 34 | 54.5 (8.2) | 36 |
| Lean body mass adjusted for height, mean (SD), kg | 38.5 (4.5) | 36 | 38.0 (3.6) | 41 |  | 55.8 (8.4) | 34 | 56.7 (5.7) | 36 |
| Fat mass, mean (SD), kg | 23.9 (11.9) | 36 | 23.8 (11.4) | 41 |  | 22.1 (9.2) | 34 | 18.7 (9.8) | 36 |
| Fat percentage, mean (SD), % | 34.7 (9.1) | 36 | 35.9 (8.5) | 41 |  | 25.9 (8.0) | 34 | 23.8 (9.8) | 36 |
| Limb/trunk fat ratio, mean (SD) | 1.1 (0.2) | 36 | 0.4 (0.05) | 41 |  | 0.7 (0.1) | 34 | 0.3 (0.04) | 36 |
| Fat mass index (SD), kg/m^2^ | 8.7 (4.1) | 36 | 9.0 (4.3) | 41 |  | 6.8 (2.8) | 34 | 6.3 (3.4) | 36 |
| Appendicular skeletal muscle mass (SD), kg | 17.1 (2.4) | 36 | 15.7 (2.3) | 41 |  | 26.6 (5.3) | 34 | 24.5 (4.4) | 36 |
| Skeletal muscle mass index (ASM/height^2^; SMI) (SD), kg/m^2^ | 5.8 (0.7) | 36 | 5.5 (0.6) | 41 |  | 7.7 (1.2) | 34 | 7.5 (1.0) | 36 |
| ASM/BMI, mean (SD), kg/(kg/m^2^) | 0.7 (0.1) | 36 | 0.6 (0.1) | 41 |  | 1.0 (0.2) | 34 | 0.9 (0.2) | 36 |

The table displays means and standard deviations for the groups.
Abbreviations: BMI, Body mass index, kg/m^2^; VLBW, very low birthweight; LBM, lean body mass; FM, fat mass; FMI, fat mass index (FM / height^2^); limb/trunk fat ratio was calculated by dividing appendicular by truncal FM; ASM, Appendicular skeletal muscle mass was calculated by subtracting appendicular bone mineral content from appendicular lean mass; SMI, Skeletal mass index (ASM/height^2^), ASM/BMI (ASM / BMI).

**eTable 3 – Differences in anthropometrics and DXA outcomes of body composition between adults born VLBW (<1500 g) and their term-born siblings.**

|  | **Model 1**^1^ | |  | **Model 2**^2^ | |
| --- | --- | --- | --- | --- | --- |
|  |  |  |  |  |  |
|  | Estimate | 95% CI |  | Estimate | 95% CI |
| **Anthropometrics** |  |  |  |  |  |
| Weight, kg | -4.52 | -7.99, -1.05 |  | -2.19 | -6.13, 1.76 |
| Height, cm | -4.54 | -6.39, -2.70 |  | -3.72 | -5.72, -1.69 |
| BMI, kg/m^2^ | -0.16 | -1.38, 1.06 |  | 0.49 | -0.90, 1.89 |
| Waist circumference | 0.06 | -3.59, 3.70 |  | 2.29 | -1.79, 6.36 |
| Hip circumference | -1.44 | -4.31, 1.43 |  | -0.04 | -3.16, 3.08 |
| Waist/hip ratio | 0.01 | -0.01, 0.03 |  | 0.02 | -0.001, 0.05 |
|  |  |  |  |  |  |
| **DXA outcomes** |  |  |  |  |  |
| Lean body mass, kg | -2.86 | -4.52, -1.20 |  | -2.02 | -3.92, -0.12 |
| Height adjusted lean body mass, kg | 0.10 | -1.18, 1.38 |  | 0.36 | -1.13, 1.85 |
| Fat mass, kg | -1.69 | -4.57, 1.20 |  | -0.26 | -3.56, 3.04 |
| Fat percentage, % | -0.36 | -2.98, 2.26 |  | 0.37 | -2.59, 3.32 |
| Limb/trunk fat ratio | -0.07 | -0.11, -0.02 |  | -0.06 | -0.11, -0.003 |
| Fat mass index, kg/m^2^ | -0.01 | -1.16, 0.98 |  | 0.35 | -0.87, 1.57 |
| Appendicular skeletal muscle mass (ASM), kg | -1.54 | -2.34, -0.73 |  | -1.22 | -2.14, -0.30 |
| Skeletal muscle mass index (ASM/height^2^; SMI), kg/m^2^ | -0.19 | -0.38, 0.01 |  | -0.14 | -0.36, 0.09 |
| ASM/BMI, kg/(kg/m^2^) | -0.05 | -0.09, -0.01 |  | -0.05 | -0.10, -0.004 |

Effect size estimates and 95% confidence intervals calculated using mixed linear regression models accounting for both preterm birth status and siblingship. A negative value represents a lower result for VLBW subjects compared to term siblings.

Abbreviations: CI, confidence interval; SD, standard deviation; ASM, Appendicular skeletal muscle mass was calculated by subtracting appendicular bone mineral content from appendicular lean mass; SMI, Skeletal mass index (ASM/height^2^), BMI, body mass index. Limb/trunk fat ratio was calculated by dividing appendicular by truncal fat mass.

^1^Model 1 adjusts for sex, and age.

^2^Model 2 additionally adjusts for maternal gestational hypertension, pre-eclampsia, and isolated proteinuria

**eTable 4 – Characteristics of study population with VLBW group subdivided into small and appropriate for gestational age (SGA and AGA).**

|  | **Term  (n = 70)^1^** | |  | **VLBW+SGA  (n = 29)** | |  | **VLBW+AGA  (n = 48)** | | **Subgroup comparison^2^** | | |
| --- | --- | --- | --- | --- | --- | --- | --- | --- | --- | --- | --- |
| Women/men | 36/34 | |  | 19/10 | |  | 22/26 | |  |  |  |
|  | Mean | SD |  | Mean | SD |  | Mean | SD | Mean difference  VLBW+SGA vs term (95%CI) | Mean difference VLBW+SGA vs VLBW+AGA  (95% CI) | Mean difference VLBW+AGA vs term  (95% CI) |
|  |  |  |  |  |  |  |  |  |  |  |  |
| Birthweight (g) | 3404 | 432 |  | 1170 | 253 |  | 1138 | 199 | -2235 (-2411; -2058) | 31 (-157; 220) | -2266 (-2416; -2058) |
| Length of gestation | 39.8 | 1.3 |  | 31.8 | 2.1 |  | 28.1 | 1.4 | -8.1 (-8.6; -7.3) | 3.6 (2.8; 4.5) | -11.7 (-12.4; -11.0) |
|  |  |  |  |  |  |  |  |  |  |  |  |
| Age (years) | 29.2 | 5.1 |  | 29.4 | 2.5 |  | 29.6 | 3.0 | -0.2 (-2.5; 2.0) | 0.2 (-1.9; 2.3) | 0.4 (-1.4; 2.2) |
| Weight (kg) | 73.9 | 17.1 |  | 63.4 | 14.2 |  | 72.5 | 15.3 | -10.5 (-18.9; -2.1) | -9.1 (-18.0; -0.2) | -1.4 (-8.5; 5.7) |
| Height (cm) | 172.6 | 9.5 |  | 162.3 | 7.2 |  | 170.6 | 9.3 | -9.9 (-14.6; -5.2) | -7.9 (-12.9; -2.8) | -2.0 (-6.0; 2.0) |
| BMI (kg/m^2^) | 24.6 | 4.5 |  | 23.9 | 5.0 |  | 24.8 | 4.6 | -0.7 (-3.1; 1.7) | -1.0 (-3.5; 1.6) | 0.2 (-1.8; 2.3) |
| Waist circumference | 84.3 | 12.2 |  | 81.7 | 14.1 |  | 85.9 | 13.6 | -4.2 (-12.3; 3.8) | -2.7 (-10.3; 4.9) | 1.6 (-4.7; 7.8) |
| Hip Circumference | 99.1 | 10.2 |  | 95.3 | 10.7 |  | 98.9 | 9.2 | -3.6 (-9.8; 2.6) | -3.7 (-9.6; 2.2) | -0.1 (-4.9; 4.6) |
| Waist/hip ratio | 0.8 | 0.07 |  | 0.9 | 0.07 |  | 0.9 | 0.09 | -0.01 (-0.05; 0.04) | 0.01 (-0.03; 0.06) | 0.02 (-0.02; 0.05) |

**eTable 4 - continued**

|  | **Term**^a^ **(n = 70)** | |  | **VLBW+SGA  (n = 29)** | |  | **VLBW+AGA  (n = 48)** | | **Subgroup comparison** | | |
| --- | --- | --- | --- | --- | --- | --- | --- | --- | --- | --- | --- |
|  | Mean | SD |  | Mean | SD |  | Mean | SD | Mean difference  VLBW+SGA vs term (95%CI) | Mean difference VLBW+SGA vs VLBW+AGA (95% CI) | Mean difference VLBW+AGA vs term  (95% CI) |
| **DXA measurements** |  |  |  |  |  |  |  |  |  |  |  |
| Lean body mass (kg) | 48.5 | 12.3 |  | 40.5 | 9.1 |  | 48.1 | 11.0 | -8.0 (-14.0; -1.3) | -7.6 (-14.0; -2.1) | -0.4 (-5.4; 4.6) |
| Fat mass (kg) | 23.0 | 10.6 |  | 21.0 | 10.2 |  | 21.7 | 11.5 | -2.1 (-7.7; 3.6) | -0,7 (-6.8; 5.3) | -1.3 (-6.1; 3.5) |
| Fat percentage (%) | 30.4 | 9.6 |  | 31.8 | 10.2 |  | 29.3 | 11.4 | 1.4 (-4.0; 6.8) | 2.5 (-3.2; 8.3) | -1.2 (-5.7; 3.5) |
| Limb/trunk fat ratio | 0.92 | 0.27 |  | 0.85 | 0.25 |  | 0.85 | 0.25 | -0.06 (-0.20; 0.07) | 0.005 (-0.14; 0.15) | -0.07 (-0.18; 0.05) |
| Fat mass index, kg/m^2^ | 7.8 | 3.6 |  | 8.0 | 4.1 |  | 7.6 | 4.2 | 0.4 (-1.7; 2.6) | 0.2 (-1.8; 2.3) | -0.2 (-1.9; 1.5) |
| Appendicular skeletal muscle mass (ASM), kg | 20.2 | 6.0 |  | 16.3 | 4.5 |  | 19.8 | 5.4 | -3.9 (-6.8; -1.0) | -3.4 (-6.5; -0.4) | -0.5 (-2.9; 2.0) |
| Skeletal muscle mass index (ASM/height^2^; SMI), kg/m^2^ | 6.7 | 1.4 |  | 6.1 | 1.2 |  | 6.7 | 1.3 | -0.6 (-1.3; 0.1) | -0.6 (1.3; 0.1) | 0.01 (-0.6; 0.6) |
| ASM/BMI, kg/(kg/m^2^) | 0.8 | 0.2 |  | 0.7 | 0.2 |  | 0.8 | 0.2 | -0.1 (-0.2; -0.02) | -0.1 (-0.2; -0.001) | -0.02 (-0.1; 0.1) |

^a^Includes two individuals born SGA.

Means and standard deviations for the groups are presented together with between-group mean differences with 95% confidence intervals.
Abbreviations: CI, confidence interval; SD, standard deviation; y, years; w, weeks; d, days; BMI, Body mass index, kg/m^2^; VLBW, very low birthweight; SGA, small for gestational age; AGA appropriate for gestational age; LBM, lean body mass; FM, fat mass; FMI, fat mass index (FM / height^2^); limb/trunk fat ratio was calculated by dividing appendicular by truncal FM; ASM, Appendicular skeletal muscle mass was calculated by subtracting appendicular bone mineral content from appendicular lean mass; SMI, Skeletal mass index (ASM/height^2^), ASM/BMI (ASM / BMI).

**eTable 5 – Differences in anthropometrics and DXA outcomes of body composition between adults born small for gestational age (SGA) at very low birth weight (VLBW, <1500 g) and their term-born siblings.**

|  | **Model 1**^1^ | |  | **Model 2**^2^ | |
| --- | --- | --- | --- | --- | --- |
|  |  |  |  |  |  |
|  | Estimate | 95% CI |  | Estimate | 95% CI |
| **Anthropometrics** |  |  |  |  |  |
| Weight, kg | -8.34 | -13.56, -3.24 |  | -5.60 | -12.49, 1.29 |
| Height, cm | -7.99 | -10.51, -5.48 |  | -7.60 | -11.00, -4.19 |
| BMI, kg/m^2^ | -0.56 | -2.32, 1.21 |  | -0.42 | -1.93, 2.78 |
| Waist circumference | -1.07 | -6.42, 4.28 |  | 1.07 | -5.51, 7.65 |
| Hip circumference | -3.25 | -7.57, 1.08 |  | -1.75 | -6.99, 3.50 |
| Waist/hip ratio | 0.02 | -0.01, 0.05 |  | 0.03 | -0.01, 0.07 |
|  |  |  |  |  |  |
| **DXA outcomes** |  |  |  |  |  |
| Lean body mass (kg) | -5.38 | -7.81, -2.94 |  | -4.88 | -8.21, -1.54 |
| Height adjusted lean body mass (kg) | -0.23 | -2.17, 1.71 |  | -0.22 | -2.88, 2.45 |
| Fat mass (kg) | -2.62 | -6.77, 1.52 |  | -0.68 | -6.22, 4.86 |
| Fat percentage (%) | -0.02 | -3.67, 3.63 |  | 1.37 | -3.47, 6.21 |
| Limb/trunk fat ratio | -0.12 | -0.19, -0.05 |  | -0.11 | -0.21, -0.02 |
| Fat mass index, kg/m^2^ | -0.11 | -1.61, 1.40 |  | 0.63 | -1.37, 2.63 |
| Appendicular skeletal muscle mass (ASM), kg | -2.58 | -3.77, -1.39 |  | -2.25 | -3.88, -0.61 |
| Skeletal muscle mass index (ASM/height^2^; SMI), kg/m^2^ | -0.31 | -0.60, -0.01 |  | -0.25 | -0.66, 0.16 |
| ASM/BMI, kg/(kg/m^2^) | -0.09 | -0.15, -0.03 |  | -0.09 | -0.17, -0.01 |

Estimates, 95% confidence intervals, and p-values calculated using mixed model regression.

Abbreviations: SGA, small for gestational age; AGA, appropriate for gestational age; CI, confidence interval; SD, standard deviation; ASM, Appendicular skeletal muscle mass was calculated by subtracting appendicular bone mineral content from appendicular lean mass; SMI, Skeletal mass index (ASM/height^2^), BMI, body mass index. Limb/trunk fat ratio was calculated by dividing appendicular by truncal fat mass.

^1^Model 1 includes dummy variables, as predictor variables, for the following three states: SGA+VLBW (i.e., variable of interest in above table), AGA+VLBW, and term born and additionally adjusts for sex, and age; ^2^Model 2 additionally adjusts for maternal gestational hypertension, pre-eclampsia, and isolated proteinuria

**eTable 6 – Differences in anthropometrics and DXA outcomes of body composition between adults born appropriate for gestational age (AGA) at very low birth weight VLBW (<1500 g) and their term-born siblings.**

|  | **Model 1**^1^ | |  | **Model 2**^2^ | |
| --- | --- | --- | --- | --- | --- |
|  |  |  |  |  |  |
|  | Estimate | 95% CI |  | Estimate | 95% CI |
| **Anthropometrics** |  |  |  | -1.18 | -5.45, 3.09 |
| Weight, kg | -2.35 | -6.39, 1.68 |  | -2.57 | -4.72, -0.43 |
| Height, cm | -2.53 | -4.57, -0.50 |  | 0.52 | -0.99, 2.02 |
| BMI, kg/m^2^ | 0.07 | -1.36, 1.50 |  | 2.67 | -1.72, 7.07 |
| Waist circumference | 0.64 | -3.54, 4.82 |  | 0.50 | -2.90, 3.89 |
| Hip Circumference | -0.55 | -3.83, 2.73 |  | 0.02 | -0.01, 0.05 |
| Waist/hip ratio | 0.01 | -0.01, 0.03 |  | -1.18 | -5.45, 3.09 |
|  |  |  |  |  |  |
| **DXA outcomes** |  |  |  |  |  |
| Lean body mass (kg) | -1.44 | -3.35, 0.47 |  | -1.20 | -3.23, 0.83 |
| Height adjusted lean body mass (kg) | 0.28 | -1.24, 1.81 |  | 0.53 | -1.09, 2.14 |
| Fat mass (kg) | -1.15 | -4.50, 2.21 |  | -0.14 | -3.70, 3.42 |
| Fat percentage (%) | -0.60 | -3.57, 2.46 |  | 0.06 | -3.10, 3.22 |
| Limb/trunk fat ratio | -0.03 | -0.09, 0.02 |  | -0.04 | -0.10, 0.02 |
| Fat mass index, kg/m^2^ | -0.08 | -1.32, 1.16 |  | 0.27 | -1.04, 1.57 |
| Appendicular skeletal muscle mass (ASM), kg | -0.95 | -1.88, -0.02 |  | -0.92 | -1.91, 0.07 |
| Skeletal muscle mass index (ASM/height^2^; SMI), kg/m^2^ | -0.12 | -0.35, 0.11 |  | -0.10 | -0.35, 0.14 |
| ASM/BMI, kg/(kg/m^2^) | -0.03 | -0.08, 0.02 |  | -0.04 | -0.09, 0.01 |

Estimates, and 95% confidence intervals calculated using mixed model regression.

Abbreviations: SGA, small for gestational age; AGA, appropriate for gestational age; CI, confidence interval; SD, standard deviation; ASM, Appendicular skeletal muscle mass was calculated by subtracting appendicular bone mineral content from appendicular lean mass; SMI, Skeletal mass index (ASM/height^2^), BMI, body mass index. Limb/trunk fat ratio was calculated by dividing appendicular by truncal fat mass.

^1^Model 1 includes dummy variables, as predictor variables, for the following three states: SGA+VLBW (i.e., variable of interest in above table), AGA+VLBW, and term born and additionally adjusts for sex, and age; ^2^Model 2 additionally adjusts for maternal gestational hypertension, pre-eclampsia, and isolated proteinuria
